# Complete Treatment Versus Residual Lesion - Long-Term Evolution After Acute Coronary Syndrome

**DOI:** 10.5935/abc.20160176

**Published:** 2016-12

**Authors:** Alexandre de Matos Soeiro, Marco Antônio Scanavini Filho, Aline Siqueira Bossa, Cindel Nogueira Zullino, Maria Carolina F. Almeida Soeiro, Tatiana Carvalho Andreucci T Leal, Carlos Vicente Serrano Jr, Ludhmila Abrahão Hajjar, Roberto Kalil Filho, Múcio Tavares Oliveira Jr

**Affiliations:** Unidade Clínica de Emergência - Instituto do Coração (InCor) do Hospital das Clínicas da Universidade de São Paulo, SP - Brazil

**Keywords:** Acute Coronary Syndrome, Treatment, Clinical Evolution, Memory, Long Term, Myocardial Infarction

## Abstract

**Introduction:**

A recently published study raised doubts about the need for percutaneous treatment of nonculprit lesions in patients with acute coronary syndromes (ACS).

**Methods:**

Retrospective, unicentric, observational study.

**Objective:**

To analyze the long-term outcomes in patients undergoing treatment of the culprit artery, comparing those who remained with significant residual lesions in nonculprit arteries (group I) versus those without residual lesions in other coronary artery beds (group II). The study included 580 patients (284 in group I and 296 in group II) between May 2010 and May 2013. We obtained demographic and clinical data, as well as information regarding the coronary treatment administered to the patients. In the statistical analysis, the primary outcome included combined events (reinfarction/angina, death, heart failure, and need for reintervention). The comparison between groups was performed using the chi-square test and ANOVA. The long-term analysis was conducted with the Kaplan-Meier method, with a mean follow-up of 9.86 months.

**Results:**

The mean ages were 63 years in group I and 62 years in group II. On long-term follow-up, there was no significant difference in combined events in groups I and II (31.9% versus 35.6%, respectively, p = 0.76).

**Conclusion:**

The strategy of treating the culprit artery alone seems safe. In this study, no long-term differences in combined endpoints were observed between patients who remained with significant lesions compared with those without other obstructions.

## Introduction

The main current guidelines on acute coronary syndromes (ACS) recommend treatment of the culprit lesion alone, except in cases with hemodynamic instability.^1-3^ Still, treatment of some significant nonculprit lesions at the time of percutaneous coronary intervention (PCI) is still controversial. Some studies have been published recently to elucidate this issue better.

Thus, there is still a knowledge gap regarding the need for percutaneous treatment of nonculprit lesions in this group of patients. The objective of this study was to evaluate the long-term outcomes in patients undergoing treatment of the culprit artery comparing those who remained with residual lesions in nonculprit arteries *versus* those without residual lesions in other coronary artery beds.

## Methods

### Study population

**Figure 1:**
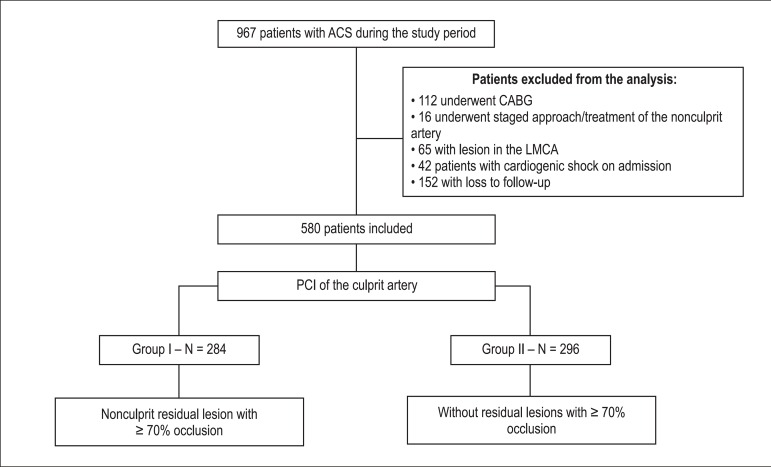
Flowchart of inclusion/exclusion of patients in the study. ACS: acute coronary syndrome; CABG: coronary artery bypass graft; LMCA: left main coronary artery; PCI: percutaneous coronary intervention.

This was a retrospective, unicentric, and observational study. We included 580 patients with ACS (with and without ST-segment elevation) admitted to an emergency service between May 2010 and May 2013. The patients were divided into two groups: group I (n = 284), with significant residual lesions (> 70%); and group II (n = 296), without residual lesions. We excluded patients who remained in clinical treatment or underwent surgical myocardial revascularization, those who underwent a staged approach at admission or treatment of nonculprit artery, and those with lesions in the left main coronary artery, cardiogenic shock, or loss to long-term follow-up (Figure 1).

We considered as having a diagnosis of ACS all patients who met the criteria established by the latest guideline of the American Heart Association.^1-3^ An ST-segment elevation ACS was defined as the occurrence of chest pain with persistent changes in the ST segment ≥ 0.1 mV in the frontal leads and ≥ 0.2 mV in the precordial leads, in at least two contiguous leads. A non-ST-segment elevation ACS was defined as the occurrence of chest pain associated with electrocardiographic changes or increase/decrease in serum troponin levels during hospitalization or, in the absence of both, clinical presentation and risk factors compatible with unstable angina (severe or increasing chest pain at rest or on minimal exertion). We considered as a reinfarction the recurrence of chest pain in association with a new elevation in serum troponin levels.

We obtained the following data: age, sex, occurrence of diabetes mellitus, hypertension, smoking, dyslipidemia, family history of early coronary disease, prior coronary artery disease (acute myocardial infarction, PCI, or prior coronary artery bypass grafting), hemoglobin, systolic blood pressure, serum creatinine level, peak serum troponin level, left ventricular ejection fraction (LVEF), number of implanted *stents*, and medications used within the first 24 hours after hospitalization.

All patients were referred to follow-up appointments at 14 days and 6 months after hospital discharge. During the appointments, ischemia tests or cardiac catheterization were performed if requested, based on a medical assessment by the team in charge of the patient. The patients were followed up through telephone contact and review of medical records. All implanted *stents* were of the conventional type and all patients maintained use of aspirin and clopidogrel for at least 12 months. Coronary reserve flow and intracoronary ultrasound were not assessed in this study.

The study was approved by the institution's research ethics committee, and all participants signed an informed consent form.

### Statistical analysis

Descriptive analyses were conducted using means, standard deviations, and minimum and maximum values. All baseline characteristics presented in Table 1 were considered as variables for the purpose of the analyses.

**Table 1:** Patients’ clinical characteristics at baseline according to allocated groups upon hospital discharge

x00A0;	Group I	Group II	p
Age	63.19 + 12.27	62.55 + 13.30	0.6
Male sex (%)	47.5%	49.5%	0.36
Diabetes mellitus (%)	33.1%	35.6%	0.23
Hypertension (%)	74.4%	81.2%	0.04
Smoking (%)	41.9%	35.1%	0.009
Positive FH for CAD (%)	15.0%	8.0%	0.02
Dyslipidemia (%)	51.9%	47.0%	0.1
Stable angina (%)	13.8%	14.5%	0.26
HF (%)	5.0%	7.1%	0.23
Prior AMI (%)	38.8%	32.3%	0.08
Prior CABG (%)	10.0%	13.8%	0.14
Prior CA (%)	30.0%	22.1%	0.05
SBP (mmHg)	132.62 + 25.56	131.67 + 25.56	0.77
Hb (g/dL)	13.83 + 1.56	13.66 + 2.10	0.36
Cr (mg/dL)	1.15 + 0.57	1.35 + 1.16	0.03
Troponin (peak) (ng/dL)	18.3 + 64.25	8.04 + 20.36	0.005
Number of stents/patient	1.41 + 0.82	1.52 + 0.74	0.37
LVEF (%)	44.59 + 22.55	41.53 + 24.00	0.04
Aspirin (%)	96.90%	96.40%	0.82
Beta-blocker (%)	80.00%	66%	< 0.001
Enoxaparin (%)	87.50%	73.20%	< 0.001
Clopidogrel (%)	58.10%	50.40%	0.06
Tirofiban (%)	10.2%	11.4%	0.42
ACEI (%)	68.10%	56.30%	0.006
Statins (%)	85.60%	79.30%	0.06

FH: family history; CAD: coronary artery disease; HF: heart failure; AMI: acute myocardial infarction; CABG: coronary artery bypass grafting; CA: coronary angioplasty; SBP: systolic blood pressure; Hb: hemoglobin; Cr: creatinine; LVEF: left ventricular ejection fraction; ACEI: angiotensin converting enzyme inhibitors.

Comparisons between groups were performed using the chi-square test for categorical variables. For continuous variables, when the Kolmogorov-Smirnov normality test showed a normal distribution, we used Student's *t* test. For non-normal distributions, the Mann-Whitney U test was used instead.

The primary outcome included combined events (reinfarction/angina, death, heart failure, and need for reintervention). The secondary outcome was mortality. The long-term analysis was performed by log-rank test to evaluate the difference between the groups in the Kaplan-Meier analysis, with a mean follow-up of 9.86 months. If any outcome differed between the groups, multivariate analysis was performed using Cox regression model. In all analyses, p values < 0.05 were considered as significant.

All calculations were performed with the software SPSS, v10.0.

## Results

The mean ages were 63 years in group I and 62 years in group II. Both groups showed significant differences regarding the prevalence of hypertension (74.4% *versus* 81.2%, p = 0.04), smoking (41.9% *versus* 35.1%, p = 0.009), and family history of coronary disease at an early age (15.0% *versus* 8.8%, p = 0.02); use of beta-blockers (80.0% *versus* 65.6%, p < 0.001), enoxaparin (87.5% *versus* 73.2%, p < 0.001), and angiotensin converting enzyme inhibitors (68.1% *versus* 56.3%, p = 0.006); baseline creatinine levels (1.15 *versus* 1.35 mg/dL, p = 0.03) and peak troponin levels (18.3 *versus* 8.04 ng/mL, p = 0.005). Table 1 shows the baseline characteristics of the study population divided by groups.

**Figure 2:**
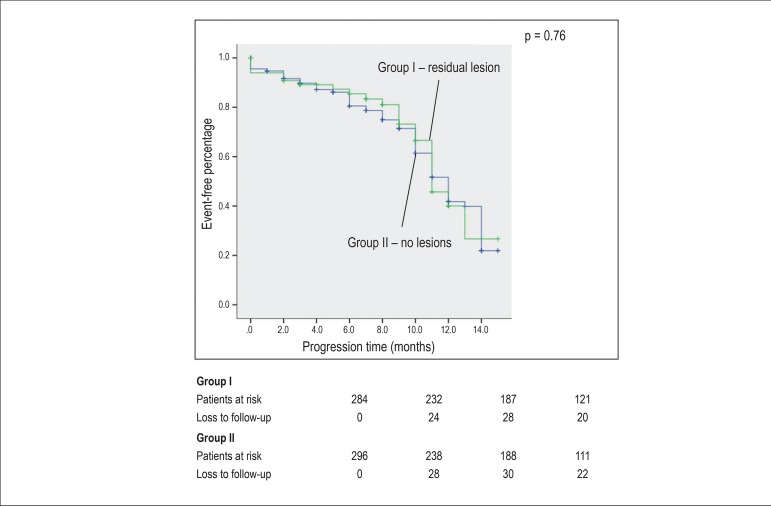
Comparison of percentage free of long-term combined events in group I (with residual lesion) and II (without residual lesion).

**Figure 3:**
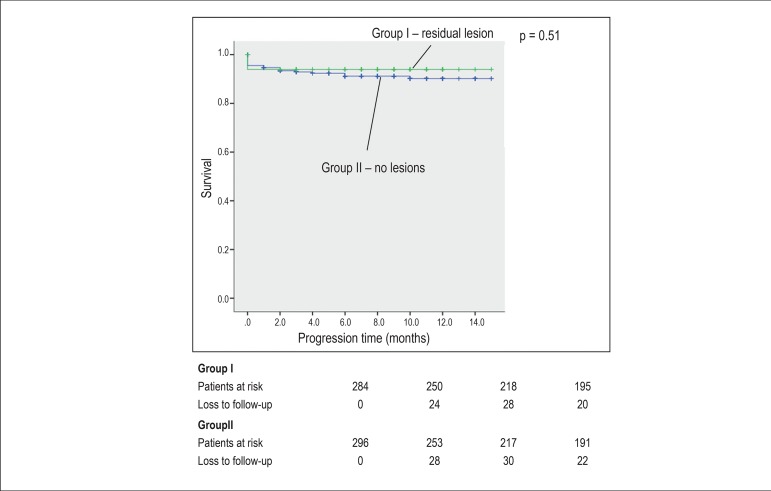
Comparison of long-term survival between group I (with residual lesion) and II (without residual lesion).

During long-term follow-up, there was no significant difference between groups I and II regarding combined events (31.9% *versus* 35.6%, respectively, p = 0.76) and mortality (6.1% *versus* 8.5%, respectively, p = 0.51) (Figures 2 and 3; Table 2).

**Table 2:** Comparative analysis of the main events between groups I (with residual lesion) and II (without residual lesion) in long-term multivariate analysis

x00A0;	Group I	Group II	p
Interventions	1.5%	0.0%	0.21
Reinfarction	15.2%	17.0%	0.44
HF	10.6%	10.1%	0.59
Mortality	6.1%	8.5%	0.51
Combined events	31.9%	35.6%	0.76

HF: heart failure.

Overall, 6.1% of the patients in group I underwent myocardial perfusion scintigraphy during follow-up, of which 38% resulted positive. In group I, 48.2% of the reinfarctions during follow-up were related to the culprit lesion in the first event and 51.8% to another lesion not addressed during the index event. In group II, these rates were 62.4% and 37.6%, respectively. In these cases, stent restenosis was observed in 28.3% and 26.5% in groups I and II, respectively. Regarding reinfarction caused by the same artery, we observed rates of 57.6% and 71.8% in groups I and II, respectively.

## Discussion

In several studies over the past 25 years, about 50% of the infarcted patients have lesions with > 50% stenosis in other coronary arteries in addition to the artery related to the infarct target artery.^1^ Aligned with information on current guidelines, the present study showed no differences in regards to long-term prognosis in patients admitted for ACS who underwent complete treatment of the coronary lesions and those who remained with residual lesions. This finding is relevant due to the recent controversy related to studies published over the past years.

The current guidelines recommend an approach dedicated to the infarct target vessel. Additional procedures, with revascularization of multiple vessels, should only be performed in cases with persistent hemodynamic instability (cardiogenic shock) or evidence of uncontrolled myocardial ischemia (pain and electrocardiographic changes). Severe coronary stenoses (> 70%) not directly related to the index procedure must be addressed at a second moment (staged procedure). In contrast, it is considered reasonable to treat severe but less complex stenoses located in the same coronary system related to the infarct vessel at the physician's discretion and before critical evaluation of the patient's clinical and hemodynamic status, including the contrast burden received by the patient.^1-3^

Corroborating the recommendations, Hannan et al.^4^ published in 2010 a database analysis aimed at comparing PCI of the culprit lesion alone (CL-PCI) *versus* PCI of all significant lesions during the index procedure (Multi-PCI) *versus* staged PCI of all significant lesions (Multi-Staged-PCI). The study included 1,434 patients with ST-elevation ACS and multivessel disease, and excluded those with lesions in the left main coronary artery, unknown LVEF, cardiogenic shock, prior myocardial revascularization, or undergoing thrombolysis. The main results obtained by the authors were lower in-hospital mortality when CL-PCI was compared with Multi-PCI (0.9% *versus* 2.4%, p = 0.04), in addition to lower 12-month mortality when CL-PCI was compared with Multi-Staged PCI (1.3% *versus* 3.3%, p = 0.04).^4^

Along with these findings, a meta-analysis published in 2014 including 39,390 patients in randomized and non-randomized studies published until October 2013 showed that the strategy of Multi-PCI compared with CL-PCI increased mortality both in the short term (odds ratio [OR] = 0.50, p = 0.002) and long term (OR = 0.52, p < 0.001).^5^

Other meta-analyses were unable to show differences among all three treatment types, raising even more doubts about the best approach to patients with ST-segment elevation ACS. In one of these meta-analyses, published in 2014, Sekercioglu et al.^6^ assessed 683 patients enrolled in randomized studies. The authors found that there was no difference between the groups in terms of overall mortality (relative risk [RR] = 0.69, 95% confidence interval [CI] 0.40 - 0.21) and mortality due to cardiac causes (RR = 0.48, 95%CI 0.22 - 1.04). It has been suggested that Multi-PCI tends to decrease those events related to revascularization; however, this observation has not shown statistically significant difference.^6^

Another meta-analysis published in 2015 and including 4,686 patients showed no difference between the groups CL-PCI and Multi-PCI in cardiac events comprising cardiac death, myocardial infarction, and revascularization within 90 days (OR = 0.70, 95%CI 0.38 - 1.27) or 1 year (OR = 0.70, 95%CI 0.47 - 1.03). There was also no difference between the groups Multi-PCI and Multi-Staged-PCI regarding cardiac events comprising cardiac death, myocardial infarction, and revascularization within 90 days or 1 year (OR = 0.86, 95%CI 0.62 - 1.08). In both comparisons, there was a decrease in revascularization rates in the group in which all arteries with lesions were either treated at the time of the PCI or underwent staged treatment, suggesting a slight benefit in these groups.^7^

An important difference between our study and the main meta-analyses discussed here is the fact that we included patients with ACS with and without ST-segment elevation. Nevertheless, we believe that in the long term, the presence of residual lesions shows correlation not with the type of ACS presentation during the index event, but with the instability of the described residual plaques.

In favor of treating all the arteries with significant lesions, Politi et al.^8^ published in 2010 a prospective randomized study with 214 patients with ST-elevation ACS and multivessel disease, which excluded patients with cardiogenic shock, prior myocardial revascularization, lesion in the left main coronary artery, and severe valvular heart disease. The objective of the study was to compare CL-PCI and Multi-Staged-PCI. A higher long-term incidence of primary compound events (reintervention, surgical revascularization, reinfarction, readmission, death due to all causes, death due to cardiac causes, in-hospital death) was observed when CL-PCI was compared with Multi-PCI and Multi-Staged-PCI (50% *versus* 20% *versus* 23%, respectively, p < 0.001). However, no difference was observed between the Multi-PCI and Multi-Staged-PCI groups.^8^

One of the first recent studies to raise doubts about the benefit of Multi-PCI treatment was the PRAMI trial, published in 2013, which included 465 patients with ST-elevation ACS and multivessel disease. This prospective, randomized, and multicenter study excluded patients with cardiogenic shock and prior revascularization. The results showed a lower incidence of the event comprising mortality from cardiac causes, refractory angina, and nonfatal reinfarction in a comparison between CL-PCI *versus* Multi-PCI (23% *versus* 9%, respectively, p < 0.001).^9^

These results were also observed in a meta-analysis published by Dahal et. al.^10^ in 2014, which included 840 patients in randomized and non-randomized studies until December 2013. The Multi-PCI and Multi-Staged-PCI strategies combined, compared with CL-PCI, decreased major cardiac events comprising myocardial infarction, revascularization, and death from all causes (RR = 0.46, 95%CI 0.35 - 0.60, p < 0.00001), mainly at the expense of myocardial infarction (RR = 0.35, 95%CI 0.17 - 0.71, p < 0.004) and revascularization (RR = 0.35, 95%CI 0.24 - 0.52, p < 0.00001). No difference occurred between simultaneous and staged treatments in regards to the occurrence of myocardial infarction (RR = 0.60, 95%CI 0.20 - 1.78, p = 0.36), revascularization (RR = 0.86, 95%CI 0.47 - 1.54, p = 0.6), and all-cause mortality (RR = 1.50, 95%CI 0.44 - 5.07, p = 0.57).^10^

Another prospective, randomized study that showed results favoring Multi-PCI was the study CvLPRIT, published in 2015, which compared CL-PCI *versus* Multi-PCI in 296 patients with ST-elevation ACS and multivessel disease. As a result, Multi-PCI showed a lower incidence of events comprising death, reinfarction, and cardiac failure at 12 months (21.2% *versus* 10%, p = 0.009).^11^

Specifically regarding Multi-Staged-PCI, one of the first studies to observe benefits with this treatment was published in 2011 and comprised a subanalysis of the Horizons study database, including 668 patients with ST-elevation ACS and multivessel disease. The results showed a lower mortality with CL-PCI when compared with Multi-Staged-PCI (9.2% *versus* 2.3%, respectively, p < 0.0001), in addition to a lower mortality from cardiac causes (6.2% *versus* 2.0%, respectively, p = 0.005) and lower rates of stent thrombosis (5.7% *versus* 2.3%, respectively, p = 0.02).^12^

In agreement with these findings, another meta-analysis with a large number of patients (46,324) published in 2014 showed no difference between the groups of CL-PCI *versus* PCI of all lesions (combining staged or simultaneous) regarding in-hospital mortality (OR = 1.11, 95%CI 0.98 - 1.25). In comparison with the Multi-Staged-PCI group, there was a decrease in in-hospital mortality in the latter (OR = 0.35, 95%CI 0.21 - 0.59). However, when the CL-PCI and Multi-PCI groups were compared, there was an increase in in-hospital mortality in the Multi-PCI group (OR = 1.35, 95%CI 1.19 - 1.54). In spite of that, in both groups with treatment of all lesions (staged and simultaneous), there was a decrease in long-term mortality (OR = 0.74, 95%CI 0.65 - 0.85) and reintervention (OR = 0.65, 95%CI 0.46 - 0.90).^13^

Finally, a recent meta-analysis (2015) also obtained similar results as the recent randomized studies cited above in a comparison between Multi-PCI *versus* CL-PCI in 775 patients. It reported a lower incidence of nonfatal infarction (3.25 *versus* 8.51%, OR = 0.376, 95%CI 0.192 - 0.763), refractory angina (4.01% *versus* 9.57%, OR = 0.400, 95%CI 0.241 - 0.741), and revascularization (10.52% *versus* 24.20%, OR = 0.336, 95%CI 0.202 - 0.661), in addition to a lower incidence of events comprising cardiac death, nonfatal infarction, and refractory angina (11.78% *versus* 28.86%, OR = 0.336, 95%CI 0.223 - 0.505).^14^

Approximately 10% to 40% of the patients included in the present study had prior coronary artery disease. This characteristic differs from most studies presented previously. If on the one hand these patients are more critically ill and have a chance of new events that will possibly increase during follow-up, on the other hand, their plaques may have more chronic features, often with evident collateral circulation and a lower chance of instability. This could justify the lack of difference in long-term events found in our study.

The present study has limitations because of its retrospective and observational design and limited sample size. In addition, differences between the groups related to LVEF, peak troponin levels, and medications used during hospitalization may interfere and modify the results, even after adjustments and multivariate analysis. However, this study presents results that are aligned with the current recommendations of ACS guidelines. Further randomized and prospective studies are still needed to clarify better this issue.

## Conclusion

The strategy of treating only the culprit artery seems safe, and in this study, showed no long-term differences in terms of combined outcomes in patients who remained with significant lesions compared with those without other obstructions.
